# Race/Ethnicity, Primary Language, and Income Are Not Demographic Drivers of Mortality in Breast Cancer Patients at a Diverse Safety Net Academic Medical Center

**DOI:** 10.1155/2015/835074

**Published:** 2015-10-28

**Authors:** Divya A. Parikh, Rani Chudasama, Ankit Agarwal, Alexandar Rand, Muhammad M. Qureshi, Taylor Ngo, Ariel E. Hirsch

**Affiliations:** Department of Radiation Oncology, Boston University School of Medicine, Boston, MA 02118, USA

## Abstract

*Objective*. To examine the impact of patient demographics on mortality in breast cancer patients receiving care at a safety net academic medical center. *Patients and Methods*. 1128 patients were diagnosed with breast cancer at our institution between August 2004 and October 2011. Patient demographics were determined as follows: race/ethnicity, primary language, insurance type, age at diagnosis, marital status, income (determined by zip code), and AJCC tumor stage. Multivariate logistic regression analysis was performed to identify factors related to mortality at the end of follow-up in March 2012. *Results*. There was no significant difference in mortality by race/ethnicity, primary language, insurance type, or income in the multivariate adjusted model. An increased mortality was observed in patients who were single (OR = 2.36, CI = 1.28–4.37, *p* = 0.006), age > 70 years (OR = 3.88, CI = 1.13–11.48, *p* = 0.014), and AJCC stage IV (OR = 171.81, CI = 59.99–492.06, *p* < 0.0001). *Conclusions*. In this retrospective study, breast cancer patients who were single, presented at a later stage, or were older had increased incidence of mortality. Unlike other large-scale studies, non-White race, non-English primary language, low income, or Medicaid insurance did not result in worse outcomes.

## 1. Introduction

Breast cancer is the most commonly diagnosed cancer and the second leading cause of cancer death among women in the United States. The National Cancer Institute estimates that there were 232,340 new breast cancer cases and 39,620 deaths in 2013 [1]. Although the advent of screening tools such as mammograms and increased public awareness surrounding breast cancer have significantly reduced the mortality associated with this disease, significant disparities in outcomes still exist [2]. Specifically, the literature has demonstrated differences in stage at diagnosis, types of treatment available, and outcomes by primary language, race, insurance type, marital status, and other demographic factors [2–4].

Disparities between Black and White women have been studied extensively. In 2013, the American Cancer Society expected the incidence of breast cancer to be 123.3 per 100,000 for White women and 118.0 per 100,000 for Black women. However, the expected breast cancer mortality rates for White and Black women were 22.4 and 31.6, respectively. Many studies postulate that these large disparities can be attributed to differences in access to care and underlying breast cancer biology [5].

Furthermore, non-English primary language may be a negative prognostic factor for breast cancer patients. Language has been shown to affect breast cancer patient treatment, yet definitive influence of language on outcomes is unclear [6]. Insurance status drives disparities in access to care and subsequently patient outcomes, in many parts of the country [7]. Several large studies have shown that women who are uninsured or have Medicaid insurance are diagnosed at later stages and have higher mortality rates than women who are privately insured [2, 8].

Across a variety of cancer types, married patients have a lower incidence and a higher rate of disease-free survival than single patients [9, 10]. Married breast cancer patients have better treatment outcomes and a lower mortality rate overall than their single counterparts [10].

Safety net hospitals are institutions that are particularly affected by health care disparities given the nature of the institution. Safety net hospitals provide a large proportion of care to the uninsured, the low-income underinsured, and Medicaid beneficiaries. These institutions represent just 2% of acute care in the United States but provide 30% of uncompensated care to most vulnerable populations. These “vulnerable” populations include the poor, ethnic minorities, non-English-speakers, substance abusers, the homeless, and individuals on public assistance programs. Many studies have shown that safety net hospitals manage patients with a greater burden of illness than higher-income populations [11]. However, despite these disparities, safety net hospitals can better meet low income patients' specialized needs related to language, culture, and transportation. Many safety net hospitals are based on coordinated care programs which involve care coordination within a single provider or system. For example, patient's visits between their primary care provider and specialists are coordinated within the same hospital system [12]. Studies have also shown that, despite caring for vulnerable and finically disadvantaged populations, safety net institutions can still achieve equal or better outcomes than non-safety net hospitals [13]. Thus, these resources show the unique environment made possible by safety net hospitals to reduce significant disparities.

Our analysis assesses disparities in breast cancer outcomes at a single institution by stratifying patients by race/ethnicity, primary language, insurance status, age at diagnosis, marital status, income, and tumor stage. As the largest safety net hospital in Massachusetts, our institution serves as a unique example of a setting with theoretically reduced barriers to healthcare access.

## 2. Patients and Methods

### 2.1. Patient Selection

A total of 1128 patients were diagnosed with breast cancer between August 2004 and October 2011 at our academic medical institution. All cases were diagnosed at our institution and received multidisciplinary care involving surgeons, medical oncologists, radiation oncologists, radiologists, and allied health professionals prior to the initiation of treatment. Eighty-two patients lacked complete demographic and tumor stage information and were excluded from the study for the missing following variables: race/ethnicity (*n* = 2), primary language (*n* = 3), insurance (*n* = 19), age (*n* = 4), income (*n* = 1), and tumor stage (*n* = 61). A retrospective analysis of 1046 patients was performed. This study was approved by our institutional review board.

### 2.2. Data Collection and Study Variables

Clinical Data Warehouse staff reviewed the institution's medical records for breast cancer patients diagnosed in the timeframe and reported their demographics and mortality status to the research team. Data included patients' self-reported information on their race/ethnicity, primary language spoken, and marital status. These demographic factors were categorized as race/ethnicity: Black, White, Hispanic, and other (Asian, Middle Eastern, Native Hawaiian, Declined to state, etc.), primary language spoken: English, Spanish, Haitian Creole, and other (Arabic, Mandarin, Hindi, Albanian, Somali, Italian, French, Vietnamese, etc.), and marital status: married, single, and other (divorced, widowed, separated, and other status). Annual average income (in US dollars) as determined by zip code was categorized as follows: ≤30,000, >30,000 to ≤50,000, >50,000 to ≤75,000, >75,000 to ≤100,000, and >100,000. Insurance status was categorized as private/commercial, charity/Medicaid/self-pay/uninsured, and Medicare/military. Cancer stage at diagnosis was classified according to the 2002 American Joint Committee on Cancer (AJCC): 0–II, III, and IV. Due to nonlinear association with mortality, age at diagnosis was categorized as ≤50, >50 to ≤60, <60 to ≤70, and >70 years of age.

### 2.3. Follow-Up and Treatment Outcomes

Breast cancer patients at our institution underwent a variety of treatments including surgery, radiation, hormone therapy, and chemotherapy. Review of medical records performed in March 2012 determined patient mortality status.

### 2.4. Statistical Analysis

Unadjusted and multivariable logistic regression models were performed to assess significant differences in mortality associated with each demographic factor. Crude and adjusted odds ratios (OR) with 95% confidence intervals (CI) were computed. All of the analyses were two-sided, and *p* values of less than 0.05 were considered statistically significant. All statistical computations were performed on SAS 9.1 system (SAS Institute, Cary, NC).

## 3. Results

### 3.1. Patient Mortality

Of 1,046 patients diagnosed with breast cancer, 120 (11.5%) died and 926 (88.5%) survived between 2004 and 2012.  Table 1 shows the descriptive statistics and mortality rate for each demographic category.

### 3.2. Race/Ethnicity and Mortality

403 (38.5%) patients were White, 374 (35.8%) were Black, 129 (12.3%) were Hispanic/Latino, and 140 (13.4%) were reported as other. There was no significant relationship between race and mortality. Black (OR = 1.01, CI = 0.54–1.87, *p* = 0.988), Hispanic (OR = 0.15, CI = 0.02–1.32, *p* = 0.088), and other (OR = 0.90, CI = 0.36–2.25, *p* = 0.822) patients were no more likely to die during the course of the study than White patients (Table 1).

### 3.3. Primary Language and Mortality

783 (74.9%) patients were principally English speakers, 94 (9.0%) were Spanish speakers, 73 (7.0%) were Haitian Creole speakers, and 96 (9.2%) spoke other languages. When compared to English speakers there was no significant difference in mortality rate between Spanish speakers (OR = 3.24, CI = 0.32–32.94, *p* = 0.320), Haitian Creole speakers (OR = 0.48, CI = 0.17–1.33, *p* = 0.159), and speakers of other languages (OR = 1.01, CI = 0.36–2.82, *p* = 0.986).

### 3.4. Insurance Type and Mortality

263 (25.1%) patients had the insurance status of private/commercial, 378 (36.1%) had Medicaid/free-care, and 405 (38.7%) had Medicare/military. When compared to private/commercial insurance status, there were no significant differences in mortality for patients with Medicaid/free-care (OR = 0.86, CI = 0.40–1.82, *p* = 0.690) and Medicare/military (OR = 1.36, CI = 0.63–2.93, *p* = 0.438).

### 3.5. Age and Mortality

**Figure 1:**
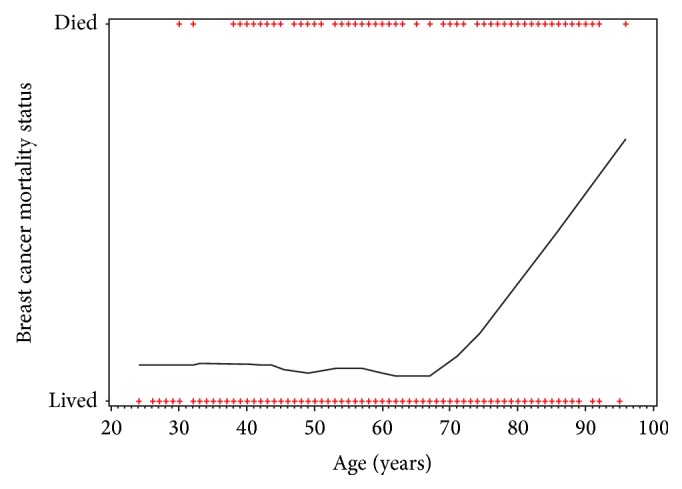
Breast cancer mortality by age shows significant increase in mortality rate after the age of 70 years.

The mean age of the patients was 59 with a standard deviation of 13.07. 70 (6.7%) patients were 40 years of age or younger, 242 (23.1%) were between 40 and 50 years of age, 298 (28.5%) patients were between 50 and 60 years of age, 229 (21.9%) patients were between 60 and 70 years of age, and 207 (19.8%) patients were older than 70 years of age. Patients who were older than 70 years of age had a significantly increased rate of mortality (OR = 3.88, CI = 1.13–11.48, *p* = 0.014) when compared to patients younger than 40 years of age. This significant increase can be seen in the graph in Figure 1. All other age groups did not have a significant relationship with mortality.

### 3.6. Marital Status and Mortality

386 (36.9%) patients were married, 370 (35.4%) were single, and 290 (27.7%) were reported as other, which included divorced, separated, widowed, or other marital status. Single patients had increased mortality (OR = 2.36, CI = 1.28–4.37, *p* = 0.006) relative to married patients. There was no significant difference in mortality between patients of other marital status when compared to married patients (OR = 1.28, CI = 0.66–2.45, *p* = 0.468).

### 3.7. Income and Mortality

Average yearly income was determined by zip code with 124 (11.9%) patients who earned less than $30,000, 548 (52.4%) patients who earned between $30,000 and $50,000, 223 (21.3%) patients who earned between $50,000 and $75,000, 84 (8.0%) patients who earned between $75,000 and $100,000, and 67 (6.4%) patients who earned more than $100,000. Income did not correlate with mortality rate for all income brackets.

### 3.8. Stage of Diagnosis and Mortality

**Figure 2:**
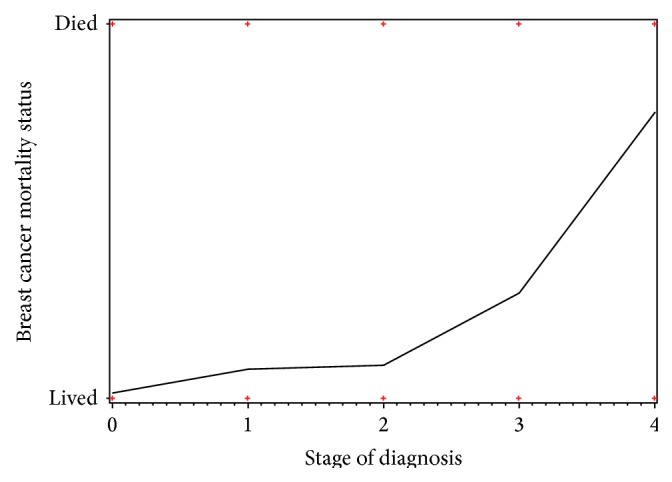
Breast cancer mortality by stage of diagnosis shows a correlation between late stage of diagnosis and increased mortality rate.

Using AJCC staging, 609 (58.2%) patients were considered at stage 0 or I, 271 (25.9%) patients were at stage II, 128 (12.2%) were at stage III, and 38 (3.6%) patients were at stage IV. When compared to patients at stage 0 or I, patients at later stages had a significantly increased rate of mortality with stage II (OR = 1.99, CI = 1.09–3.61, *p* = 0.024), stage III (OR = 12.26, CI = 6.57–22.89, *p* < 0.0001), and stage IV (OR = 171.81, CI = 59.99–492.06, *p* < 0.0001). This correlation can be seen in Figure 2.

## 4. Discussion 

Since 1970s, the American Cancer Society has reported disparities in cancer incidence and mortality with an emphasis on race and socioeconomic status [14]. In a study conducted at two public hospitals in Chicago, IL, with over 1,200 patients, Ansell et al. reported that, from 1973 to 1985, Black women with breast cancer had a lower 5-year breast cancer survival rate and higher overall mortality rate than their White counterparts [15]. However, there is a national trend suggesting the declining impact of race on mortality [16]. Newman et al. conducted a meta-analysis of 20 studies between 1980 and 2001, with a total of over 90,000 patients, to assess the impact of race on outcomes after adjusting for age, stage, and socioeconomic status. The authors found that there was a 22% excess risk of death for Black patients with breast cancer. However, when including data up to 2005, this percentage dropped to 19%, suggesting a decline in racial disparities [5]. Furthermore, Bradley et al. in 1929 showed that, after controlling for Medicaid enrollment and poverty, there was no difference in outcomes between Black and White women [8]. Our analysis supports the trend of reduction of racial disparities in breast cancer mortality because, after adjusting for insurance type, income, and other demographic factors, there was no significant relationship between race and mortality in our patient population.

Past studies have indicated that lower income and insurance status are drivers of disparity in patient diagnosis and mortality. In a study conducted by McGinnis et al. of 191,714 non-Hispanic White patients diagnosed with breast cancer between 1995 and 1996, 12.1% of patients from low income areas were initially diagnosed at stage III or IV compared to 10.0% of patients from high income areas [14]. Women in census tracts that had lower incomes were more likely to have a later stage of diagnosis, receive inadequate treatment, and have poor survival [17]. Poverty, as measured by Medicaid status and census data, continues to be a risk factor for breast cancer diagnosis, treatment, and death [18]. Freedman et al. reviewed data of 662,117 patients from National Cancer Data base hospitals between 1998 and 2005 and showed that uninsured patients, Medicaid enrollees, and Medicaid beneficiaries, when compared to privately insured patients, had lower odds of receiving adequate treatment when compared to privately insured patients [19].

Despite the history of disparities in cancer outcomes based on income and insurance status, these factors had no significant impact on cancer mortality in our study. This may be due to our institution's use of patient navigation services. Patient navigation services have emerged as a potential solution for improving cancer care delivery [20]. As listed on our institution's website, “Patient navigation services are comprised of people from the same communities as patients, who are specially trained to help patients overcome their barriers to care. Patient navigators assist patients with transportation needs, help schedule appointments, provide linkages to community and hospital services and sometimes, just sit with patients and listen to them as they share their fears and feelings about their illness.” This peer patient navigation service utilizes members from the patients' communities to teach them how to face challenges linked to income barriers and to access appropriate insurance benefits. Navigation services have been shown to bridge low socioeconomic status patients' hospital and community lives, which may otherwise be at odds [21]. Thus, combining our institution's role as a safety net hospital and patient navigation services, financial and insurance status disparities are reduced.

Our institution's role as a safety net hospital could have also helped to reduce many disparities. Our center is a not for profit hospital with its mission to provide consistently accessible health services to all and 75% of our institution's patient visits come from underserved populations. With a focus on urban health, our institution has established an integrated health care delivery system with a network affiliation of a medical center and 14 community centers. As a safety net institution, our center could better meet our patient population's needs related to language, culture, and transportation through the services mentioned above.

Language did not affect patient mortality in this study. Language barriers can hinder communication between physician and patient, which can affect treatment utilization and, consequently, patient prognosis. Karliner et al. surveyed over 300 oncologists and surgeons in California to determine that at least 91% of physicians reported using patients' family members or friends as interpreters. These doctors acknowledged difficulty in discussing treatment options and prognosis with non-English speaking patients. Meanwhile, physicians who used professional interpreters reported that they were more likely to have patient-centered-treatment discussions in which to explain risks and benefits of treatment options [6]. Our institution offers robust professional interpreter services that involve in-person, video, and phone interpreters. Specifically, as seen on our institution's website, in addition to in-person interpreters in 15 spoken languages, American Sign Language, and Certified Deaf Interpreting, our center utilizes telephonic and video interpreting in order to have constant service in 240 languages. Interpreter services may have reduced the language barriers in access to care as well as mortality outcomes in breast cancer patients.

Other than stage at diagnosis and age, single marital status was the only demographic factor that was significantly associated with increased mortality. Married patients generally enjoy overall better health and increased life expectancy [10]. Married patients with cancer have 15% reduced mortality when compared to unmarried patients. This survival advantage may be due to spousal encouragement of healthy lifestyle and social support. Married patients are also more likely to be diagnosed at an earlier stage. Aizer et al. analyzed over 1 million cancer patients to assess the impact of marital status at diagnosis, use of therapy, and cancer specific mortality. Single patients presented more often with metastatic cancer, received inadequate treatment, and had increased mortality [9]. Single women may decline therapies, such as axillary dissection or radiation, due to insufficient postoperative care or transportation [10]. Christie et al. found that single women reported much higher depression scores than partnered women with breast cancer [22]. Giese-Davis et al. analyzed 125 women with metastatic breast cancer to show that decreased depressive symptoms over the first year after receiving treatment predicted better survival [23]. Thus, it may be helpful for healthcare providers to assess patients' existing social support networks and offer ways to build or use other social groups. Improving patient support systems could alter treatment outcomes for single patients [10, 22].

This study has several limitations. Annual income, as determined by the average income for the zip code, does not necessarily indicate the actual income status of a patient. The study does not address comorbid medical conditions. It is also limited to a single institution that is a safety net hospital which may differ in terms of demographics and patient population from other non-safety net hospitals. Further research should seek to better understand demographic disparities in breast cancer outcomes to better apply disparity-eliminating strategies nationwide. Research into programs' offering social support systems to patients may decrease the marriage mortality gap. Interpreter and patient navigation services in healthcare should be implemented and evaluated for efficacy on a larger scale.

In conclusion, there appears to be a trend showing a decline in demographic disparities on breast cancer mortality. Analyzing disparity-targeted programs may elucidate the decreasing disparities and barriers to care.

## Figures and Tables

**Table 1:** Risk of breast cancer mortality by demographic factors.

Characteristic	*N* (1046)	Deaths *n* (%)	Unadjusted OR^*∗∗*^ (95% CI)	*p* value	Multivariable OR^*∗∗*^ (95% CI)	*p* value
Race						
White	403	49 (12.2)	1.00^*∗*^		1.00^*∗*^	
Black	374	51 (13.6)	1.14 (0.75–1.74)	0.539	1.01 (0.54–1.87)	0.988
Hispanic	129	7 (5.4)	0.42 (0.18–0.94)	0.035	0.15 (0.02–1.32)	0.088
Other	140	13 (9.3)	0.74 (0.39–1.41)	0.359	0.90 (0.36–2.25)	0.822
Primary language						
English	783	96 (12.3)	1.00^*∗*^		1.00^*∗*^	
Spanish	94	6 (6.4)	0.49 (0.21–1.15)	0.100	3.24 (0.32–32.94)	0.320
Haitian Creole	73	8 (11.0)	0.88 (0.41–1.89)	0.745	0.48 (0.17–1.33)	0.159
Other	96	10 (10.4)	0.83 (0.42–1.66)	0.601	1.01 (0.36–2.82)	0.986
Insurance type						
Commercial/private	263	17 (6.5)	1.00^*∗*^		1.00^*∗*^	
Medicaid/free-care	378	36 (9.5)	1.52 (0.84–2.77)	0.169	0.86 (0.40–1.82)	0.690
Medicare	405	67 (16.5)	2.87 (1.64–5.00)	0.0002	1.36 (0.63–2.93)	0.438
Age (years)						
<40	70	8 (11.4)	1.00^*∗*^		1.00^*∗*^	
>40 to ≤50	242	19 (7.9)	0.66 (0.28–1.58)	0.351	0.63 (0.23–1.73)	0.369
>50 to ≤60	298	24 (8.1)	0.68 (0.29–1.58)	0.370	0.51 (0.18–1.42)	0.195
>60 to ≤70	229	15 (6.6)	0.54 (0.22–1.34)	0.186	0.72 (0.24–2.23)	0.575
>70	207	54 (26.1)	2.74 (1.23–6.08)	0.014	3.88 (1.13–11.48)	0.014
Marital status						
Married	386	33 (8.6)	1.00^*∗*^		1.00^*∗*^	
Single	370	49 (13.2)	1.63 (1.02–2.60)	0.039	2.36 (1.28–4.37)	0.006
Other	290	38 (13.1)	1.61 (0.99–2.64)	0.058	1.28 (0.66–2.45)	0.468
Income (US dollars)						
≤30,000	124	21 (17.0)	1.00^*∗*^		1.00^*∗*^	
>30,000 to ≤50,000	548	58 (10.6)	0.58 (0.34–1.00)	0.050	0.59 (0.29–1.20)	0.145
>50,000 to ≤75,000	223	25 (11.2)	0.62 (0.33–1.16)	0.134	0.41 (0.17–1.03)	0.057
>75,000 to ≤100,000	84	6 (7.1)	0.38 (0.15–0.98)	0.045	0.61 (0.20–1.86)	0.383
>100,000	67	10 (15.0)	0.86 (0.38–1.95)	0.720	0.95 (0.33–2.75)	0.928
Stage of diagnosis						
0, I, IA	609	31 (5.1)	1.00^*∗*^		1.00^*∗*^	
II, IIA, IIB	271	24 (8.9)	1.81 (1.04–3.15)	0.035	1.99 (1.09–3.61)	0.024
III, IIIA, IIIB, IIIC	128	36 (28.1)	7.30 (4.30–12.37)	<0.0001	12.26 (6.57–22.89)	<0.0001
IV	38	29 (76.3)	60.08 (26.18–137.85)	<0.0001	171.81 (59.99–492.06)	<0.0001

*n*: number of patients. ^*∗*^Reference category. ^*∗∗*^Odds ratios were calculated using logistic regression.
